# Corrected analysis of “the effects of bright light treatment on affective symptoms in people with dementia: a 24-week cluster randomized controlled trial” that accounts for clustering and nesting verifies conclusions

**DOI:** 10.1186/s12888-023-05180-2

**Published:** 2023-09-20

**Authors:** Yasaman Jamshidi-Naeini, Lilian Golzarri-Arroyo, Abu Bakkar Siddique, Colby J. Vorland, Miriam Jocelyn Rodriguez, Richard J. Holden, David B. Allison

**Affiliations:** 1 Department of Epidemiology and Biostatistics, Indiana University School of Public Health-Bloomington, Bloomington, IN USA; 2 School of Public Administration, Florida Atlantic University, Boca Raton, FL USA; 3 Department of Health and Wellness Design, Indiana University School of Public Health-Bloomington, Bloomington, IN USA; 4 Indiana University Center for Aging Research, Regenstrief Institute, Inc, Indianapolis, IN USA; 5 Center for Health Innovation and Implementation Science, Indiana University, Indianapolis, IN USA

## Abstract

In this correspondence, we explain the reasoning for invalidity of the analysis choices by Kolberg et al., and provide the results produced using correct statistical procedures for their study design. Reassuringly, we could verify the original conclusions. That is, results of the corrected statistical models are similar to the results of the original analysis. Regardless of the magnitude of difference that corrected statistical methods make, results and conclusions that are derived from invalid methods are unsubstantiated. By verifying the results, we allow the readers to be assured that the published conclusions in the study by Kolberg et al. now rest on a sound evidential basis.

Kolberg et al. conducted a cluster-randomized controlled trial (cRCT) to study the effects of Bright Light Treatment (BLT) on behavioral and psychological symptoms of dementia (BPSD) [1] in patients residing in nursing homes. In this cRCT, 8 nursing homes (i.e., clusters) were randomized to either the control or the BLT groups. The published results indicate statistically significant effects of BLT compared to control on mood-related and affective symptoms. Kolberg et al. [1] did not account for clustering effect of the nursing homes (which induces a pattern of correlated data) and nesting (i.e., individuals nested in nursing homes, which limits degrees of freedom for testing the intervention effect) in their analyses.

Clustering and nesting have implications for statistical inference in cRCTs and must be taken into account. In cRCTs, participants within the same cluster potentially have common experiences and characteristics. Thus, “errors” (model residuals) are not independent. Ignoring this potential dependency within clusters (i.e., clustering, most commonly quantified by Intraclass Correlation Coefficient) can result in misestimation of the components of variance [2, 3]. Additionally, degrees of freedom must be adjusted during statistical analysis of cRCTs because “the units of observation (i.e., individual participants) are nested within the units of assignment (i.e., clusters)” [4]. This limits the sample size, and thus, degrees of freedom for accurate estimation of the intervention effect [3]. Results and conclusions derived from statistical tests that ignore clustering and nesting are unreliable.

The authors confirmed our understanding about their statistical analytic choices, and collegially provided access to the deidentified raw data underlying the published results in their paper. In the original study, the R package lme4 was used and random intercepts for individual patients were included in the multilevel linear regression models. This adjusts for correlation due to repeated measurements on each subject, but not for clustering. We re-analyzed the data using the package lme4 multilevel linear regression models including random intercept for individuals nested within nursing homes to account for clustering, and random intercept for individuals to account for the repeated measures. We also adjusted for degrees of freedom to account for the nesting effect of the design.

The p-values of both analyses’ results are shown in Table 1. Reassuringly and similar to the results of the original analysis, results of the corrected statistical models indicate statistically significant time-by-group interaction effects on mood-related and affective symptoms (i.e., BPSD). Thus, through reanalyzing the data using valid methods, we could verify the original conclusions.

**Table 1:** P-values of time-by-group interaction effects from multilevel linear regression models with and without taking clustering and nesting into account

	Outcome variables presented in Table 6 and Table 7 of the original study	P-values of time-by-group effect in the models that do not account for clustering and nesting (original models)	P-values of time-by-group effect in the models that account for clustering and nesting (corrected models)
Cornell Scale for Depression in Dementia	Total	0.092	0.108
	Mood-related signs	**0.003**	**0.006**
	Behavioral disturbance	0.356	0.387
	Cyclic functions	0.544	0.550
Neuropsychiatric Inventory - Nursing Home Version	Total	0.107	0.125
	Affective symptoms	**0.020**	**0.030**
	Psychosis	0.477	0.485
	Agitation	0.658	0.652

Regardless of the magnitude of difference that corrected statistical methods make, results and conclusions that are derived from invalid methods are unsubstantiated. That is, unless verified through reanalysis, unsubstantiated results should not be utilized and relied upon by readers. Kolberg et al.’s study addresses a critical need in nursing home settings by providing evidence to support a potentially viable treatment for BPSD among dementia patients. Using rigid statistical approaches is essential for the clinical implications of the study and ensure optimally informed and evidence-based treatment decisions. By our verifying the results, we allow the readers to be assured that the published conclusions in the study by Kolberg et al. now rest on a sound evidential basis.

For upholding the integrity of science, it is vital for reported results and conclusions to be generated using valid statistical procedures for the study design. That is, the validity of statistical methods is strictly a function of pre-specifiable required conditions derived from theory, and these required conditions do not vary with empirical context. In contrast, the robustness of statistical methods can depend on empirical factors [5], which may vary by field. Scholars in and outside of aging, dementia, and sleep research are advised to incorporate and report clustering and nesting adjustments in their analyses of cRCTs. When this is not done, it is advisable to share data for others to apply and report on corrected statistical analyses of these data to verify or correct conclusions.

## Data Availability

Statistical code is available at https://osf.io/avb2k/. Dr. Flo-Groeneboom (the senior author of 10.1186/s12888-021-03376-y) provided us with temporary remote access to the dataset used for this re-analysis. We did not have any special access privileges that others would not have.

## Abbreviations

BLT: Bright Light Treatment
cRCT: cluster Randomized Controlled Trial
BPSD: Behavioral and Psychological Symptoms of Dementia
